# The impact of patella baja on the knee joint: A case report

**DOI:** 10.1016/j.radcr.2025.06.071

**Published:** 2025-07-17

**Authors:** Zineb Yammouri, Soukaina Beyyato, Hajar Ouazzani Chahdi, Ismail Chaouche, Amal Akammar, Nizar El Bouardi, Meriem Haloua, Moulay Youssef Alaoui Lamrani, Meryem Boubbou, Mustapha Maaroufi, Badreddine Alami

**Affiliations:** a Department of Adult radiology, CHU Hassan II Fez, Sidi Mohammed Ben Abdellah University, Fez, Morocco; b Department of Mother and Child radiology, CHU Hassan II Fez, Sidi Mohammed Ben Abdellah University, Fez, Morocco

**Keywords:** Patella baja, Knee joint function, Patellofemoral biomechanics, MRI, Conservative management

## Abstract

Patella baja, or patella infera, is a condition characterized by an abnormally low patellar position relative to the femur, significantly affecting knee joint mechanics and function. This condition can result from congenital factors, postsurgical changes, trauma, or degenerative diseases. Patella baja leads to increased patellofemoral joint stress, reduced quadriceps efficiency, and restricted knee mobility, contributing to pain and functional impairment. Diagnosis relies on imaging techniques such as X-ray, MRI, CT scan, and ultrasound, which help assess patellar height, soft tissue integrity, and joint alignment. Treatment options range from conservative approaches, including physical therapy, bracing, and anti-inflammatory medications, to surgical interventions such as patellar tendon lengthening, tibial tubercle osteotomy, and arthroscopic debridement. Early diagnosis and appropriate management are essential to prevent long-term complications and improve patient outcomes.

## Introduction

Patella baja, also known as patella infera, refers to an abnormally low position of the patella relative to the femoral trochlea, which alters normal knee biomechanics. The condition may be congenital or, more commonly, acquired, particularly following knee surgeries such as total knee arthroplasty (TKA) or anterior cruciate ligament (ACL) reconstruction. Other contributing factors include trauma, chronic inflammatory conditions, and prolonged immobilization [[1], [2], [3]].

This inferior patellar positioning leads to increased patellofemoral contact pressure, reduced quadriceps efficiency, and restricted joint motion, resulting in anterior knee pain, stiffness, and potentially early degenerative changes [4,5].

Diagnosis is primarily radiographic, using lateral knee X-rays and validated indices such as the Insall-Salvati ratio or Caton-Deschamps index to objectively assess patellar height. MRI and ultrasound complement this evaluation by providing detailed visualization of tendon morphology and cartilage status [1,3,5].

This case report describes a patient with symptomatic patella baja, highlighting the clinical presentation, imaging characteristics, and treatment approach. It aims to increase awareness of this underrecognized source of anterior knee dysfunction and underscore the importance of timely diagnosis and individualized management.

## Case presentation

A 40-year-old white woman presented with a 1-year history of chronic anterior knee pain that progressively limited her daily activities, including stair climbing and prolonged walking. The pain was localized to the right knee and described as deep, aching, and persistent, without episodes of locking or instability. The patient denied any history of prior surgical procedures on the affected limb but recalled a mild traumatic incident 1 year earlier while descending stairs. The injury was initially managed conservatively with rest and nonsteroidal anti-inflammatory drugs, but without significant improvement.

On physical examination, there was tenderness along the patellar tendon and the inferior pole of the patella, accompanied by mild quadriceps atrophy and limited active extension. No signs of joint effusion or ligamentous laxity were observed.

**Fig. 1:**
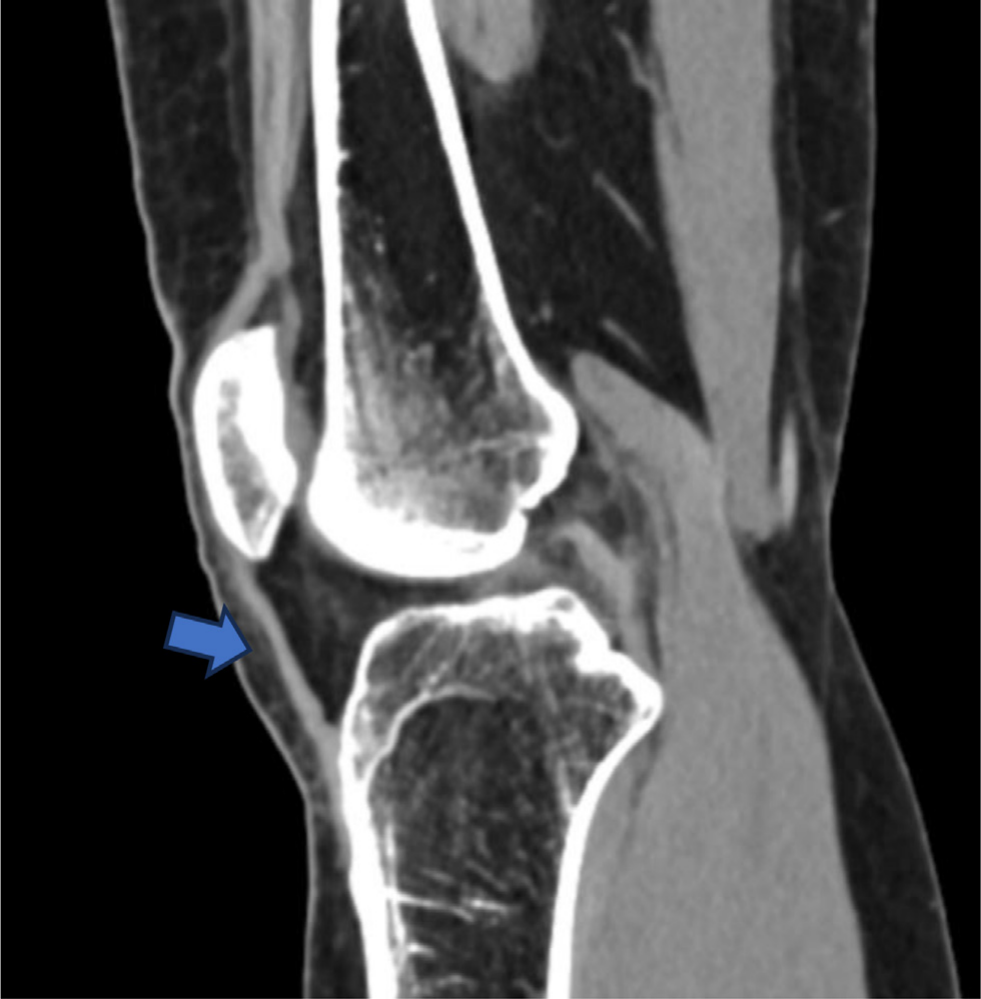
Sagittal CT image of the right knee (bone window) Sagittal computed tomography (CT) image showing a low-riding patella in relation to the femoral trochlea, consistent with patella baja. Blue arrow indicates the shortened patellar tendon.

**Fig. 2:**
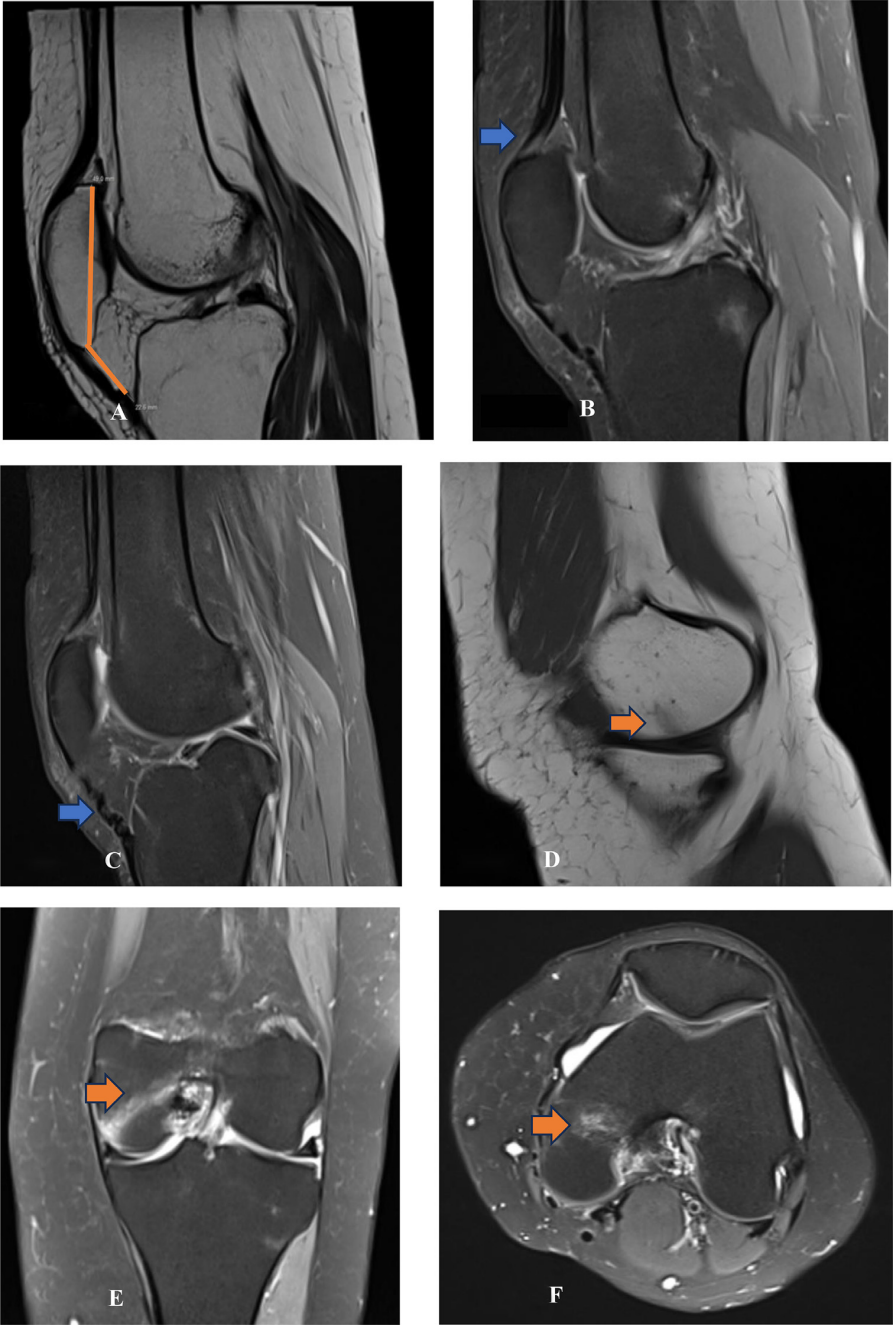
Multiplanar MRI views of the right knee (A) Sagittal T1-weighted image: Shows low patellar position with an Insall-Salvati ratio of 0.5. The patellar tendon appears thickened and tortuous. (B) Sagittal DP-weighted image: Demonstrates mild quadriceps tendinopathy, with signal alteration at the tendon insertion (blue arrow). (C) Sagittal T1-weighted image: Demonstrates a tortuous appearance of the patellar tendon (blue arrow), consistent with chronic tendinopathy. (D) Sagittal T1-weighted image: Reveals a linear subchondral fissure in the medial femoral condyle (orange arrow), suggesting focal bone injury. (E–F) Coronal DP-weighted (E) and axial T1-weighted (F) images: Reveal bone marrow edema in the medial femoral condyle (orange arrows), consistent with subchondral stress response on multiple planes.

Given the chronicity of symptoms and their functional impact, imaging studies were obtained. CT and MRI examinations revealed a low-riding patella. The Insall-Salvati ratio was measured at 0.5, consistent with patella baja. Additional findings included shortening and thickening of the patellar tendon, moderate quadriceps tendinopathy, and early chondral degeneration of the patellofemoral joint Fig. 1, Fig. 2.

The patient was started on a structured physical therapy program focusing on quadriceps strengthening, hamstring flexibility, and patellar mobilization. In the absence of prior surgery and given the early stage of degenerative changes, conservative management was prioritized. She was also counseled on activity modification and prescribed a patellar unloading brace.

At the 3-month follow-up, the patient reported modest improvement in both pain and function, although discomfort persisted during high-demand activities. Surgical options—including tibial tubercle osteotomy and patellar tendon lengthening—were discussed but deferred in favor of continued nonoperative management and periodic reassessment.

## Discussion

This case offers a unique opportunity to explore the functional, structural, and therapeutic implications of patella baja in a nonsurgical setting, as discussed across biomechanical, pathological, diagnostic, and therapeutic perspectives.

### Biomechanical considerations

The patella functions as a fulcrum within the knee’s extensor mechanism, optimizing the mechanical advantage of the quadriceps during extension [3]. In patella baja, the abnormally low position of the patella shortens the lever arm, resulting in reduced quadriceps efficiency and increased patellofemoral joint stress. These biomechanical changes impair functional activities such as stair climbing and rising from a seated position. Increased contact stress within the patellofemoral joint has also been quantitatively demonstrated in postarthroplasty settings, further supporting the mechanical burden imposed by patella baja [4]. In our patient, these deficits were clinically evident, with reduced quadriceps strength and impaired functional mobility correlating with a markedly reduced Insall-Salvati ratio of 0.5.

### Pathophysiology and degenerative risk

By increasing patellofemoral contact pressure, patella baja predisposes the joint to chondral damage and early-onset osteoarthritis [6]. It also restricts patellar tendon excursion, potentially reducing knee flexion range [5]. Chronic overload and inflammation likely contributed to the tendon thickening and cartilage irregularities seen on MRI in this case. Muscle imbalances and altered load distribution may also play a role in the pathogenesis of tendon and cartilage degeneration in such cases [10].

### Etiology and epidemiological context

Although patella baja is most frequently encountered following knee surgeries such as total knee arthroplasty (TKA) or anterior cruciate ligament (ACL) reconstruction [6,7], it can also result from trauma, fractures, prolonged immobilization, or chronic inflammatory diseases [8,9]. While postoperative origins are predominant in the literature, nonsurgical causes—such as the mild trauma reported in our patient—should not be overlooked. This context highlights the need for clinical vigilance, especially in patients with a history of injury but no surgical intervention.

### Diagnostic approach

Accurate and timely diagnosis is essential in patients with unexplained anterior knee pain. Patellar height assessment is typically based on radiographic indices such as the Insall-Salvati ratio (normal ≥0.8), complemented by the Caton-Deschamps (normal ≥0.6) and Blackburne-Peel (normal ≥ 0.8) ratios [1,3,11]. These indices can also be applied to MRI and CT. In our patient, the significantly reduced Insall-Salvati ratio (0.5) confirmed the diagnosis. MRI provided detailed insights into soft tissue pathology, including quadriceps tendinopathy and early cartilage degeneration, while CT ruled out bony malalignment and deformities [1,3,12,13]. Imaging findings aligned closely with the patient’s clinical symptoms, reinforcing the diagnostic value of multimodal imaging in nonsurgical patella baja.

### Management considerations

Conservative treatment—comprising physical therapy, activity modification, and patellar unloading bracing—is typically effective in early or moderate cases [13]. When structural damage is limited, nonoperative management may delay or even eliminate the need for surgery. Surgical options such as tibial tubercle osteotomy or patellar tendon lengthening are reserved for patients with persistent symptoms and functional limitations despite conservative care [14]. Regardless of the approach, structured rehabilitation is critical to restoring function and preventing recurrence [15].

This case underscores the need to recognize atypical, nonsurgical presentations of patella baja. Multimodal imaging proved essential in clarifying the diagnosis and guiding a successful conservative strategy. Such cases emphasize the importance of individualized, imaging-guided treatment plans in optimizing patient outcomes and avoiding unnecessary surgical intervention.

## Conclusion

Patella baja significantly alters knee biomechanics by increasing patellofemoral stress and reducing the efficiency of the extensor mechanism. While it is most commonly associated with prior knee surgeries, this case illustrates an atypical, nonpostoperative presentation following minor trauma—highlighting the need to broaden the differential diagnosis in patients with chronic anterior knee pain.

The markedly reduced Insall-Salvati ratio (0.5), along with MRI findings of tendon shortening and early chondral degeneration, underscores the value of advanced imaging in identifying structural abnormalities even in the absence of surgical history. This case demonstrates how combining radiographic indices with multimodal imaging can guide appropriate, conservative management and help avoid premature surgical intervention.

Ultimately, this report provides new insight into the effective nonoperative management of early-stage patella baja and reinforces the importance of individualized evaluation and timely diagnosis in optimizing functional outcomes.

## Patient consent

I, the author of the article *«* The Impact of Patella Baja on the Knee Joint: a case report*»,* declare that the patient provided permission to publish the details of his case and for the future use and publication of his images, at the time the images were obtained.
